# Assessing Orthorexia Nervosa: Validation of the Polish Version of the Eating Habits Questionnaire in a General Population Sample

**DOI:** 10.3390/nu12123820

**Published:** 2020-12-14

**Authors:** Anna Brytek-Matera, Natalija Plasonja, Greg Décamps

**Affiliations:** 1 Faculty of Psychology in Katowice, SWPS University of Social Sciences and Humanities, 40-326 Katowice, Poland; 2 LabPsy, University of Bordeaux, F-33000 Bordeaux, France; natalija.plasonja@u-bordeaux.fr (N.P.); greg.decamps@u-bordeaux.fr (G.D.)

**Keywords:** orthorexia nervosa, eating behaviors, body dissatisfaction, general population sample, exploratory factor analysis, confirmatory factor analysis, multigroup confirmatory factor analysis

## Abstract

Extreme focus on healthy eating, called orthorexia nervosa (ON), was assessed using a 21-item Eating Habits Questionnaire (EHQ). The present study aimed to validate the Polish version of the EHQ in a general population sample. Nine hundred sixty-seven women (59%) and men participated in the present study. Data was obtained from an internet-administered survey. Exploratory factor analysis with the first split sample (*n* = 502) produced a three-factor solution accounting for 47% of the variance. In confirmatory factor analysis with the second split sample (*n* = 465), the three-factor structure showed satisfactory goodness-of-fit (comparative fit index (CFI) = 0.99, root mean square error of approximation (RMSEA) = 0.008). Reliability analysis for the Polish version of the EHQ across the whole questionnaire showed strong internal consistency (α = 0.88, intraclass correlation coefficient (*ICC*) = 0.86). The internal consistency, measured by Cronbach’s alpha coefficients, for the EHQ subscales were 0.85 (knowledge), 0.81 (problems), and 0.81 (feelings and behaviors). Total EHQ score was positively correlated with its three subscales, cognitive restraint, uncontrolled eating, and emotional eating, and was negatively correlated with body mass index (BMI). The Polish version of the EHQ is a reliable questionnaire that can be used with confidence to better assess ON in a general population sample.

## 1. Introduction

Attitudes towards healthy eating and dietary behavior have changed in western societies in recent years. Increasing attention is being paid to eating high-quality food [1]. The desire to eat healthy foods is not in itself a disorder [2]; however, restrictive eating patterns may contribute to increasing health risks [3]. An extreme adherence to clean eating (characterized by proper nutrition, restrictive eating patterns, and strict avoidance of foods considered to be unhealthy or impure) [1] may result in a pathological fixation with healthy eating, called orthorexia nervosa (ON) [4]. ON is defined as an obsession, a fixation, or preoccupation with healthy food consumption. The concern about healthy diet results in the attention captured by food (preoccupation); thus, evolving to a persistent and disturbing thought (obsession) and a stereotyped behavior (fixation) [5]. ON is related to the attempt to achieve optimal health by paying attention to healthy dietary intake [6]. Four classification approaches suggest the diagnostic criteria for ON [7,8,9,10] which refer to: (1) Obsessional or pathological preoccupation with “healthy”, “pure”, or “clean” foods, (2) rigid avoidance of foods considered “unhealthy” or “unclean”, (3) emotional consequences for transgressing self-imposed dietary rules (distress at violation of food rules/distress when violating food rules), and (4) impairment to social, physical, and/or psychological wellbeing resulting from these food beliefs and behaviors [5,11]. It is worth noting that ON has so far not been recognized either by the Diagnostic and Statistical Manual of Mental Disorders (DSM-5) or by the International Classification of Diseases (ICD 11). This may be explained by the still ongoing discussion about the conceptualization of ON and its categorization among other mental disorders (a distinct disorder, a variant of eating disorder, or a variant of obsessive–compulsive disorder). Previous studies have shown that ON is related to disordered eating attitudes [5,12,13], eating disorders symptoms (e.g., dietary restraint, shape and weight concern) [14,15], body dissatisfaction [16,17], as well as obsessive–compulsive disorder symptoms [18,19].

Despite the lack of a universally accepted definition or diagnostic criteria, ON has been studied progressively in the last two decades, which has resulted in an increased amount of ON measures. To the best of our knowledge, there are thirteen distinct ON assessment tools (in alphabetical order): The Barcelona Orthorexia Nervosa Scale [20], the Body-Image Screening Questionnaire (BISQ) [21], Burda-Orthorexia Risk Assessment (B-ORA) [22], Bratman Orthorexia Test (BOT) [23], the Düsseldorf Orthorexia Scale (DOS) [24], the Eating Habits Questionnaire (EHQ) [25], the Eating Habits Questionnaire-Revised (EHQ-R) [26], the Orthorexia Nervosa Inventory (ONI) [13], the Orthorexia Nervosa Scale (ONS) [27], the ORTO-15 [28], the ORTO-R [29], the Scale to Measure Orthorexia in Puerto Rican Men and Women [30], and the Teruel Orthorexia Scale (TOS) [31]. The detailed characteristics of ON tools are presented in the recent systematic review by Opitz et al. [32], showing that existing measures demonstrate questionable psychometric properties (BOT, ORTO-15), challenge preliminary diagnostic criteria (DOS, TOS), and require further evaluation (e.g., EHQ-R, ONS, B-ORA). No gold standard exists for the assessment of ON so far [32]. Nevertheless, the recent study [33] found that BOT, EHQ, and DOS are internally reliable self-report instruments (a unidimensional structure—higher ordered for the EHQ, good internal reliability) and their high intercorrelations (*r*s > 0.70) support convergent validity, indicating that they essentially measure the same construct. In contrast, the ORTO-15 was found to have an unacceptable model fit, low internal reliability, and medium-sized correlations with the other three ON questionnaires, suggesting that it measures a different construct and/or captures ON tendencies less precisely [33]. Thus, according to Meule et al. [33], research on ON should preferably use the BOT, EHQ, or DOS. To have more solid evidence, other researchers [13,34] are also considering using the EHQ and DOS questionnaires, which seem to be reliable assessments of ON symptomatology.

Although the ORTO-15 [28] has been the most widely used tool to assess ON worldwide [35], the EHQ has increasingly been used. The EHQ [25] evaluates cognitions (knowledge of healthy eating), behaviors (problems associated with healthy eating), and feelings (feeling positively about healthy eating) towards an extreme focus on healthy eating. The conceptualization of ON, on which the EHQ is based, defines it as an “overwhelming” preoccupation about eating healthfully. To the best of our knowledge, a total of seventeen studies [36,37,38,39,40,41,42,43,44,45,46,47,48,49,50,51,52] have used the EHQ. This questionnaire has been translated into Italian [46] and French [42] so far. Australian validation of the EHQ also exists [43]. However, the EHQ has never been validated in a Polish population. The EHQ is an already established tool with promising psychometric properties [43] (except for the lack of criterion-related validity [47], and no criticisms have been raised towards EHQ. Therefore, this study aimed to validate the Polish version of the EHQ in a general population sample. In addition, we investigated the association between ON, eating behaviors (cognitive restraint, uncontrolled eating, emotional eating), and body dissatisfaction in a large general population sample of Polish adults.

## 2. Materials and Methods

### 2.1. Participants and Study Design

A cross-sectional population-based survey was conducted on 967 Polish women (59%) and men (41%). The mean age was 23.35 (*SD* = 4.96; age range: 18–60 years). The average body mass index (BMI) was 22.5 kg/m^2^ (*SD* = 3.78, BMI range: 16.2–38.1 kg/m^2^). The response rate was 19.42%.

The survey was administered via the internet and recruited a convenience sample. Participants received notice about the research (through social media platforms) with the announcement including the online link to the study. Interested individuals were invited to visit a website that directed them to the consent form, information form (purpose of the current study, anonymity, voluntariness of consent to research), and questionnaires. All participants offered their informed consent before starting the survey (by ticking a respective box at the first page of the online survey) and responded voluntarily to the survey. There was no financial compensation for participating in the study. 

The current study is part of a large project focusing on the impact of negative affect on eating behavior. The research project (Harmonia 10) was funded by the National Science Centre (NCN), Poland (Grant no. 2018/30/M/HS6/00022). The Harmonia 10 research project was approved by the Research Ethics Committee at the Institute of Psychology, University of Wroclaw, Poland (no. IPE 0019). All procedures performed in our study were in accordance with the 1964 Helsinki Declaration (adopted by the 18th World Medical Association General Assembly, Helsinki, Finland) and its later amendments or comparable ethical standards.

### 2.2. Outcome Measures

#### 2.2.1. The Eating Habits Questionnaire (EHQ)

The EHQ [25] assesses the cognitions, behaviors, and feelings regarding an extreme focus on healthy eating, called ON. It consists of 21 items scored on a 4 points Likert-type scale (1: False, not at all true; 4: Very true) and assembled in three different factors: Knowledge of healthy eating (5 items), problems associated with healthy eating (12 items), and feeling positively about healthy eating (4 items). The EHQ shows good internal consistency (Cronbach’s α = 0.82, 0.90, and 0.86, respectively) [25]. In the present study, the Polish translation of the EHQ [37] was used (Appendix A). A high score on this scale portrays higher ON tendencies.

#### 2.2.2. The Three-Factor Eating Questionnaire (TFEQ-R18)

The TFEQ-R18 [53] assesses eating behaviors. It consists of 18 items scored on a 4-point response scale (1: Definitely true; 4: Definitely false) and is arranged into three different aspects of eating behavior: Cognitive restraint (i.e., conscious restriction of food intake in order to control body weight or to promote weight loss; 6 items), uncontrolled eating (i.e., tendency to eat more than usual due to a loss of control over intake accompanied by subjective feelings of hunger; 9 items), and emotional eating (i.e., inability to resist emotional cues, the tendency to eat in response to negative emotions; 3 items). In the present study, we used the Polish version of the TFEQ-R18 [54], which has demonstrated satisfactory levels of internal reliability (α = 0.78 for cognitive restraint, α = 0.84 for uncontrolled eating, and α = 0.86 for emotional eating). Higher scores are indicative of greater cognitive restraint, uncontrolled eating, and emotional eating.

#### 2.2.3. The Body Dissatisfaction (BD) Scale

The BD assesses discontentment with the overall shape and size (e.g., “I think my hips are too big”). It is a subscale of the Eating Disorder Inventory (EDI) [55], the most widely used multidimensional self-report instrument that evaluates the presence of eating disorder psychopathology. It consists of 9 items on a 6-point response scale (1 = Never; 6 = Always). Higher scores on the scale indicate a greater dissatisfaction with one’s body. In the present study, we used the Polish version of the BD scale [56], which has shown a satisfactory internal consistency (α = 0.92).

#### 2.2.4. Anthropometric and Demographic Measures

In addition to completing the aforementioned questionnaires, the participants reported their age, gender, weight, and height, the latter two allowing us to calculate their body mass index (BMI).

### 2.3. Statistical Analysis

In order to investigate the factorial structure of the Polish version of the EHQ, the sample was randomly split into two for exploratory factor analysis (EFA) and confirmatory factor analysis (CFA).

Both multivariate and univariate normality of the data were examined using Mardia’s coefficient and the Shapiro–Wilk test, respectively. Normality was lacking if Mardia’s coefficient |*z*| score exceeded 5 and if the Shapiro-Wilk test was significant (*p* < 0.05) [57]. All the statistical analyses were performed using the R version 3.4.2. for Windows.

#### 2.3.1. Exploratory Factor Analysis (EFA)

The EFA was carried out on a first randomly split sample. Prior to the analysis, the correlations between the items as well as the Kaiser–Meyer–Olkin index (KMO) and the Bartlett sphericity index were calculated in order to investigate the adequacy of the data for an EFA. The significant values of the correlation coefficients and the Bartlett index (*p* < 0.05) as well as the satisfactory value of the KMO coefficient (*KMO* ≥ 0.70) indicated that the data were appropriate for an EFA.

The factor extraction method was chosen according to whether the distribution of the data followed the normal distribution [57]. The number of the factors retained was established using the parallel analysis (eigenvalue > 1) as well as the Cattell criterion [58]. Finally, items that presented nonsignificant correlations with other items, loaded on two or more factors, and/or had low factor loadings (<0.40) were removed from the factorial solution.

#### 2.3.2. Confirmatory Factor Analysis (CFA)

Once the exploratory factor analysis was completed, an independent confirmatory factor analysis was conducted on the other randomly split sample using the lavaan package [59]. The model’s goodness of fit was analyzed according to the following criteria: *χ*^2^/df, comparative fit index (CFI), Tucker–Lewis index (TLI), adjusted goodness-of-fit index (AGFI), root mean square error of approximation (RMSEA), and standardized root mean square (SRMR). The fit was considered satisfactory when *χ*^2^/df was less than 5, when CFI, TLI, and AGFI were superior to 0.90, and when RMSEA and SRMR were inferior to 0.8, with values inferior to 0.6 being considered as excellent [60].

#### 2.3.3. Multigroup Confirmatory Factor Analysis

The measurement invariance of the Polish version of the EHQ across participants’ gender (female vs. male) was explored using a multigroup confirmatory factor analysis with lavaan [59] and semTools [61] packages. Four different models were tested for this purpose. Model 1 tested for configural invariance (testing the hypothesized model across the two gender categories, without constraints). Model 2 tested for metric invariance (factor loadings were constrained to equality across gender). Model 3 tested for scalar invariance (factor loadings and item intercepts were constrained to equality across the two gender categories). Finally, model 4 tested for strict measurement invariance (factor loadings, item intercepts, and item error variances were constrained to equality across gender). Measurement invariance of the models was supported if ∆*χ*^2^ was not significant, ∆CFI ≤ 0.005, ∆RMSEA ≤ 0.01, and ∆SRMR ≤ 0.025 when testing for loadings invariance, and if ∆CFI ≤ 0.005, ∆RMSEA ≤ 0.01 and ∆SRMR ≤ 0.005 when testing for intercept and residual invariance [62]. Before comparing these four models, it was verified that the configural model had an acceptable fit. For this purpose, the aforementioned CFA criteria were applied. Following the inspection of the configural model, measurement invariance was explored by comparing the four models with each other.

#### 2.3.4. Reliability and Sensibility Analyses

The reliability of the Polish version of the EHQ scale was explored using the split-half method and the Cronbach’s alpha coefficient (α). The reliability of the scale was considered to be good if the interclass correlation coefficient, measured between even and odd items, was greater than 0.70 [63,64]. The internal consistency of the scale was interpreted based on the following Cronbach’s alpha criteria: α < 0.60—unacceptable; 0.60 > α > 0.70—correct; 0.70 > α > 0.80—satisfactory; 0.80 > α > 0.90—very good; α > 0.95—problematic [65].

In order to study the scale’s sensibility, Ferguson’s *δ* coefficient and discrimination analysis of items were performed. Ferguson’s *δ* coefficient expressed the extent to which the scale discriminated between individuals and ranged from 0 to 1. A delta close to 0 indicated that all subjects had the same score, while a delta close to 1 indicated that each subject had a different score [66]. The discrimination analysis of items relied on the following criteria: Items with *d* < 0.20 were poor and needed to be revised or eliminated; items with *d* of 0.20–0.29 were marginal and needed revision; items with *d* of 0.30–0.39 were reasonably good but could be improved; and items with *d* > 0.40 were very good [67].

#### 2.3.5. Relationships with Sociodemographic Variables

Nonparametric Spearman’s *rhô* correlation coefficients were calculated between the Polish version of the EHQ, age, BMI, body dissatisfaction, and the three subscales of the TFEQ-R 18. Mean score comparisons based on participants’ gender were conducted employing the non-parametric Wilcoxon–Mann–Whitney U tests. Cohen’s *d* coefficient was used to evaluate the size effect of the differences. According to Cohen [68], a size effect equal to 0.20 was considered small, a *d* = 0.50 was regarded as a medium effect, and *d* = 0.80 was considered to be a large size effect.

## 3. Results

### 3.1. Exploratory Factor Analysis (EFA)

The EFA was conducted on a first randomly split sample (*n* = 502). The Mardia’s coefficient *z* score was greater than 5 (|*z*| = 119.080, *p* < 0.001), indicating that the multivariate normality condition was not met, as well as the univariate normality of the data, supported by a significant Shapiro–Wilk test (*p* < 0.001). Hence, nonparametric statistics were employed for the Polish validation of the EHQ.

The Bartlett’s sphericity test and the Kaiser–Meyer–Olkin index (*KMO*) had correct values (Bartlett test: *χ*^2^(210) = 4472.741, *p* < 0.001; *KMO* = 0.92), indicating that the data were appropriate for the EFA. The factor extraction method used was the principal axis method, which did not require a normal distribution of the data [57], and the oblimin rotation was applied.

According to the correlation matrix, all items were significantly correlated with each other. The Cattell index and the Kaiser criterion (eigenvalue > 1), obtained using a parallel analysis, suggested a 3-factor solution. While exploring the factorial solution of the scale, two different models were tested. Initially, six items (items 3, 4, 7, 10, 15, and 20) were removed from the first model because they loaded significantly on two factors. However, after conducting an AFC on both gender groups, the indexes revealed that the model fit was poor in the male sample (CFI and TLI values were below 0.90). Therefore, considering the low *R*^2^ (*R*^2^ = 0.182) of item 16 in the male group, as well as the modification indices, item 16 was excluded from the second and final model, and the correlation between the errors from the items 6 and 8 as well as 8 and 18 were added. The remaining 14 items explained 47% of the total variance. The factors correlated moderately with each other. The correlation between F1 and F2 was 0.34, between F2 and F3 0.36, and between F1 and F3 0.59. The results from the EFA are presented in Table 1.

### 3.2. Comfirmatory Factor Analysis (CFA)

The CFA was carried out on the second randomly split sample (*n* = 465) using the MLR estimator. The results presented in Table 2 revealed a good adjustment of the model.

**Figure 1:**
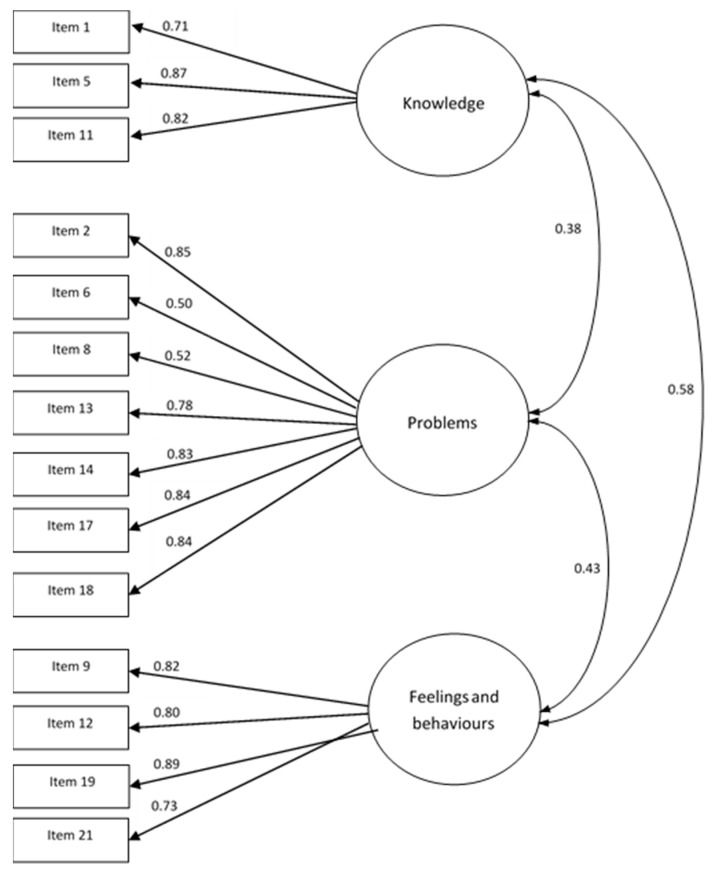
Standardized factor loadings of the three-factor model for the total sample after confirmatory factor analysis.

Considering the content of each subscale, the terms “knowledge” (knowledge of healthy eating) incorporating items 1, 5, and 11, “problems” (problems associated with healthy eating) including items 2, 6, 8, 13, 14, 17, and 18, and “feelings and behaviors” (feeling positively about healthy eating and behaviors associated with healthy eating) composed of items 9, 12, 19, and 21 were given to the three subscales of the Polish version of the EHQ (Figure 1).

### 3.3. Multigroup Confirmatory Factor Analysis

Multigroup confirmatory factor analyses were conducted next in order to explore the EHQ’s measurement invariance across gender (female vs. male).

The 967 participants were divided in two groups according to their gender. Thus, the female group was composed of 573 women (*M*_age_ = 23.86, *SD* = 5.77 years) and the male group was composed of 394 men (*M*_age_ = 22.62, *SD* = 3.34 years).

First, a regular CFA was performed separately on each of the two gender groups. The fit indexes of the model tested in the two groups were satisfactory (Table 3).

Next, four different models of measurement invariance were explored. The comparison of the configural and metric measurement invariance models reported the following indexes: ∆CFI = 0.022; ∆SRMR = 0.003; ∆RMSEA = 0.01. Even though the ∆CFI was higher than the cut-off value of 0.05, we decided to pursue the analysis, given the fact that the values of the other two indexes were correct and the ∆*χ*^2^ was not significant (∆*χ*^2^ = 38.93, *p* > 0.05).

The comparison between models 3 and 2 showed that the additional constraint did not significantly weaken the fit (∆CFI = 0.001; ∆SRMR = 0.000; ∆RMSEA = 0.002; ∆*χ*^2^ = 7.696, *p* > 0.05). Finally, the last two models’ comparison (models 4 and 3) argued that the added constraint did not significantly alter the fit (∆CFI = 0.002; ∆SRMR = 0.003; ∆RMSEA = 0.002; ∆*χ*^2^ = 9.619, *p* > 0.05). These results demonstrate that the factor structure, the factor loadings, as well as the intercepts and measurement errors, were equivalent according to gender.

### 3.4. Reliability and Sensibility Analyses

Cronbach’s alpha coefficient and the split-half method were performed in order to explore the reliability of the Polish version of the EHQ. The intra-class coefficient with a Spearman–Brown correction, calculated between even and odd items, was equal to 0.86. The values of the Cronbach’s alpha coefficients were 0.85 for “knowledge”, 0.81 for “problems”, 0.81 for “feelings and behaviors”, and 0.88 for the whole scale. Important correlations between items, as well as good Cronbach’s alpha coefficient values, implied a satisfactory reliability of the Polish version of the EHQ. Ferguson’s *δ* coefficient was 0.98 and the discrimination analysis of the items was satisfactory with the discrimination index being superior to 0.40 for all the items. These results supported the hypothesis of the adequate reliability and sensibility of the Polish version of the EHQ scale.

### 3.5. Relationships with Sociodemographic Variables

Since the data was not distributed normally, a non-parametric Spearman’s *rhô* correlation coefficient was calculated in order to explore the links between different scales. All the correlations are displayed in Table 4.

BMI was correlated with EHQ total score (*rhô* = −0.06) and the “knowledge” subscale (*rhô* = −0.08). The total EHQ score and its three subscales were strongly correlated with each other. Positive correlations were observed between the total EHQ score and cognitive restraint (*rhô* = 0.32), uncontrolled eating (*rhô* = 0.10), and emotional eating (*rhô* = 0.11). While the “problems” and “feelings and behaviors” scales both presented positive correlations with body dissatisfaction (*rhô* = 0.14 and *rhô* = 0.1, respectively), uncontrolled eating (*rhô* = 0.11), emotional eating (*rhô* = 0.10 and *rhô* = 0.12, respectively) and cognitive restraint (*rhô* = 0.24 and *rhô* = 0.30, respectively), the “knowledge” scale was only positively related to cognitive restraint (*rhô* = 0.24).

Non-parametric Wilcoxon–Mann–Whitney U tests were conducted in order to compare the scores from different scales according to participants’ gender. There was a significant difference in the EHQ total score and “feelings and behaviors” across gender: Women had higher scores than men (*p* < 0.001). However, the size effect of those differences was quite small: *d* = 0.14 and *d* = 0.17, respectively. Significant differences across gender were also observed in the mean scores of the cognitive restraint, uncontrolled eating, and emotional eating subscales. The results revealed that men had higher sores on these three variables (*p* < 0.001). The size effect of these differences was moderate: *d* = −0.54, *d* = −0.75 and *d* = −0.43, respectively. The results are presented in Table 5.

## 4. Discussion

The objective of the present study was to investigate the psychometric properties of the Polish version of the EHQ in a general population sample. The CFA yielded a shorter version composed of 14 items (items 1, 2, 5, 6, 8, 9, 11, 12, 13, 14, 17, 18, 19, 21) for which the three-factor solution showed satisfactory goodness-of-fit (Table 2). Compared to the original scale, the Polish validation encompassed the same number of factors but a reduced number of items (14 items instead of 21). The Polish version of EHQ also had a similar factorial structure to the French validation [42]. However, the number of items was not the same: 5 items were removed from the French version [42], while 7 items were removed from the Polish validation. Furthermore, those two versions had only two removed items in common, items 15 and 16. However, the content of their subscales were quite similar. The subscale “positive feeling of control” from the French version [42] embodied all the items from the “feelings and behaviors” subscale of the Polish validation. Likewise, the “problems of attention control” and “social relation” from the French version [42] consisted of almost all the items from the “problems” subscale of the Polish validation. Apart from the same two items being deleted from the French [42] and Polish validations, there were no other similarities relating to the withdrawn items between the different EHQ validations. In addition, no statistical parameter could explain the removal of the items from the Polish validation. Nevertheless, the majority of the items removed from the Polish validation belonged to the “problems” subscale of the original version. Hence, the withdrawal of those specific items may have been due to the ambiguity of the item’s content in Polish.

The EHQ has been validated in Italian [46] and French populations [42] so far. Novara et al. [46] investigated the psychometric properties of the Italian version of the EHQ among a non-clinical female sample (*n* = 204). Results from EFA and CFA (Table 2) revealed that the 21-item Italian version of the EHQ [46] represented three ON factors: Knowledge of healthy eating (knowledge), problems associated with healthy eating (problems), and feeling positively about healthy eating (feelings). These findings were consistent with the original three dimensions of the EHQ. The Italian version of the EHQ total score correlated with the Eating Disorder Inventory-3-Referral Form (EDI-3-RF). The psychometric properties of the EHQ were also investigated in a large sample of French adults (*n* = 2065) [42]. Results of the CFA revealed a very good fit of the improved measurement model (Table 2). A 16-item EHQ [42] represented three ON dimensions: Rigid eating behavior (eating behavior characterized by the rigid pursuit of a strict diet with many rules; REB), positive feeling of control (feeling of one’s capacity to maintain a healthy diet involving a positive feeling of control; PFC), and problems of attention control and social relationships (difficulty to control attention and to maintain the quality of social relationships because of intrusive thoughts related to the obsession of healthy eating; PACSR). The reliability analysis showed that REB (α = 0.82), PFC (α = 0.76), and PACSR (α = 0.75) measurements had satisfactory internal consistency [42]. The reliability and validity of the EHQ was also examined on Australian adult women (*n* = 286) [43]. The EFA established that the EHQ represented four ON dimensions: Healthy eating cognitions, dietary restriction, diet superiority, and social impairment. Cronbach’s α coefficient was 0.89 for the total EHQ scale and ranged from 0.72 to 0.80 for the four subscales [43] Criterion-related validity showed a significant moderate to strong negative correlation between the EHQ and ORTO-10 (a ten-item version of ORTO-15) [43].

Reliability analysis for the Polish version of the EHQ across the whole questionnaire showed strong internal consistency (α = 0.88; *ICC* = 0.86). The internal consistency of the EHQ subscales, measured by Cronbach’s alpha coefficients, was 0.85 for “knowledge” (knowledge of healthy eating), 0.81 for “problems” (problems associated with healthy eating), and 0.81 for “feelings and behaviors” (feeling positively about healthy eating and behaviors associated with healthy eating). These values were similar to those reported in other validations, i.e., alpha values between 0.75 and 0.90 [25,42,43].

Our results demonstrated that the total EHQ score was positively correlated with its three subscales (“knowledge”, “problems”, and “feelings and behaviors”), cognitive restraint, uncontrolled eating, and emotional eating, as well as negatively correlated with BMI. This suggests that EHQ score was associated with eating behaviors related to, on the one hand, the intention to control food intake in order to maintain or lose weight and, on the other hand, to consuming more than usual due to a loss of control over intake, accompanied by subjective feelings of hunger and the tendency to eat in response to negative emotions. When cognitive control was undermined, dietary restraint led to reduced sensitivity to internal cues for satiety and, therefore, resulted in overeating [69]. In the case of ON (using the EHQ), preoccupation with healthy food consumption may be related to decreasing sensitivity to internal cues for satiety and may have an effect on uncontrolled eating (e.g., consumption of foods considered to be unhealthy or impure) or emotional eating. The total EHQ score as well as the “feelings and behaviors” and “problems” subscales were positively correlated to cognitive restraint, body dissatisfaction, uncontrolled, and emotional eating, while the “knowledge” subscale was only correlated to cognitive restraint. The absence of significant correlations between the “knowledge” scale and body dissatisfaction, uncontrolled, or emotional eating may be explained by the fact that individuals who scored high on the “knowledge” scale were satisfied with their body, did not have a problematic eating behavior, and were restraining their food intake in order to keep a healthy weight and avoid health consequences related to overweight and obesity. These findings support those of Depa et al. [70], which have identified the motivations for weight control as being involved in orthorexic behavior. The authors explained that, related to the orthorexic behavior, weight control can reduce or prevent health consequences engendered by overweight and obesity.

Our results were consistent with the recent study [42] revealing the negative association between two ON dimensions (REB and PFC) and BMI. In addition, this study noticed that people with high levels of ON symptoms did not typically present high or low BMI, which indicated that ON dimensions were only marginally related to BMI [42]. Our results were in contrast to a previous study [50] that found no relationship between ON (measuring by the EHQ) and BMI, and another one [47] that showed that high BMI was associated with greater ON symptomatology for men (high-BMI and low-BMI women were at equal risk for ON). It is worth pointing out that research investigating the link between ON and BMI have reported inconsistent results. The majority of studies noticed that both high and low BMI are risk factors for developing ON [71].

Additionally, our findings showed that women had higher scores on the EHQ total score and on the “feelings and behaviors scale” when compared to men. However, the size effect of those differences was quite small (*d* = 0.14 and *d* = 0.17, respectively). Findings regarding the gender differences in ON were inconsistent. The recent meta-analysis on gender differences in ON [72] pointed out that men represented higher normative healthy eating behaviors and showed more problems from this rigid eating behavior, whereas pathologically healthful eating behaviors among women were more linked with having positive feelings about their healthy eating [72].

A strength of our study was the use of a large population-based sample, representative of the Polish general population in terms of age and gender. Our study had some limitations. First, the cross-sectional design did not allow for the assessment of test–retest reliability of the questionnaire. Then, due to the self-report nature of the data, the results may have been susceptible to a common method and social desirability biases. Although the risk was reduced by relying on anonymity and assuring voluntary participation, it was impossible to completely eliminate the problem. Finally, a selection bias was present because of the convenience sampling technique followed.

The objective of the present was to validate the Polish version of the EHQ in a general population sample. Future study should evaluate the reliability and consistency of the EHQ in more specific clinical groups (e.g., individuals with ON, individuals with ED) as well as in non-clinical groups (e.g., adolescents). Adolescents may be at high risk for disordered eating behavior. The previous study [73] has shown that ON prevalence in the adolescent population was similar to that found in the adult population.

## 5. Conclusions

Our findings suggest that the Polish version of the EHQ has adequate psychometric properties for measuring ON in a large population-based sample. Therefore, it is possible to use the Polish version of the EHQ for applications in research and clinical practice.

## Figures and Tables

**Table 1:** Exploratory factor analysis (EFA) results of the Polish version of the EHQ.

EHQ Items	Factor Loading		
	F1	F2	F3
1. I am more informed than others about healthy eating.	**0.53**	−0.03	0.21
2. I turn down social offers that involve eating unhealthy food.	0.13	**0.49**	0.05
3. The way my food is prepared is important in my diet.	0.4	0.03	0.42
4. I follow a diet with many rules.	0.31	0.13	0.42
5. My eating habits are superior to others.	**0.83**	−0.07	0.08
6. I am distracted by thoughts of eating healthy.	−0.16	**0.56**	0.17
7. I only eat what my diet allows.	0.4	0.37	−0.02
8. My healthy eating is a significant source of stress in my relationships.	−0.12	**0.79**	−0.07
9. I have made efforts to eat more healthily over time.	−0.07	0.03	**0.73**
10. My diet affects the type of employment I would take.	0.2	0.44	0
11. My diet is better than other peoples’ diets.	**0.79**	0.07	−0.02
12. I feel in control when I eat healthily.	0.11	0.09	**0.64**
13. In the past year, friends or family members have told me that I’m overly concerned with eating healthily.	0.15	**0.62**	−0.01
14. I have difficulties finding restaurants that serve the foods I eat.	0.13	**0.58**	−0.05
15. Eating the way I do gives me a sense of satisfaction.	0.2	−0.01	0.39
16. Few foods are healthy for me to eat.	−0.07	0.51	0.14
17. I go out less since I began eating healthily.	0.09	**0.65**	−0.03
18. I spend more than three hours a day thinking about healthy food.	−0.1	**0.74**	0.09
19. I feel great when I eat healthily.	−0.02	−0.07	**0.79**
20. I follow a health-food diet rigidly.	0.37	0.43	0.09
21. I prepare food in the most healthful way.	0.16	0.18	**0.47**

Items in bold were included in the final Polish version of the Eating Habits Questionnaire (EHQ).

**Table 2:** Goodness-of-fit indices of the Polish version of the EHQ (PL-EHQ) and its comparison with the original version of the EHQ, as well as with the Italian and French versions of the EHQ.

EHQ and Its Versions	*χ* ^2^	*p*	*χ* ^2^ *df*	RMSEA	SRMR	AGFI	TLI	CFI
EHQ [18]	NA	NA	NA	0.07	NA	NA	0.90	0.91
PL-EHQ	76.240	0.406	1.030	0.008	0.056	0.983	0.999	0.999
Italian version of the EHQ [46]	228.190	0.020	NA	0.050	NA	NA	NA	0.990
French version of the EHQ [42]	NA	NA	NA	0.04	0.05	NA	0.98	NA

NA—information not reported in the article; EHQ—Eating Habits Questionnaire; PL-EHQ—the Polish version of the EHQ; RMSEA—root mean square approximation; SRMR—standardized root mean square; AGFI—adjusted goodness-of-fit index; TLI—Tucker–Lewis index; CFI—comparative fit index.

**Table 3:** Goodness-of-fit indices of models testing for cross-gender invariance.

Model	CFI	SRMR	RMSEA	*χ* ^2^	ΔCFI	ΔSRMR	ΔRMSEA	Δ*χ*^2^
Female	0.945	0.042	0.046	160.881				
Male	0.925	0.050	0.044	126.038				
Configural	0.935	0.045	0.046	289.515				
Metric	0.957	0.048	0.036	250.585	0.022	0.003	0.01	38.93
Scalar	0.959	0.048	0.034	258.281	0.001	0	0.002	7.696
Strict	0.961	0.051	0.032	267.9	0.002	0.003	0.002	9.619

CFI—comparative fit index; SRMR—standardized root mean square; RMSEA—root mean square approximation.

**Table 4:** Correlations for study variables.

Variables	Age	BMI	BD	EHQ	K	P	F&B	UE	EE
1. Age									
2. Body mass index	0.22 *								
3. Body dissatisfaction (EDI)	0.01	0.18 *							
4. Eating Habits Questionnaire total score	−0.05	−0.06 *	0.11 *						
5. Knowledge (EHQ)	0	−0.08 *	−0.01	0.76 *	-				
6. Problems (EHQ)	−0.07 *	−0.04	0.14 *	0.81 *	0.38 *				
7. Feelings and behaviors (EHQ)	−0.04	−0.04	0.1 *	0.83 *	0.58 *	0.43 *			
8. Uncontrolled eating (TFEQ-R18)	−0.16 *	0.04	0.11 *	0.1 *	0	0.11 *	0.11 *		
9. Emotional eating (TFEQ-R18)	−0.1 *	0.12 *	0.14 *	0.11 *	0.03	0.1 *	0.12 *	0.74 *	
10. Cognitive restraint (TFEQ-R18)	−0.02	0.1 *	0.08 *	0.32 *	0.24 *	0.24 *	0.3 *	0.33 *	0.34 *

BMI—body mass index, EDI—Eating Disorder Inventory; BD—Eating Disorder Inventory—Body Dissatisfaction Scale; EHQ—Eating Habits Questionnaire; TFEQ-R18—Three-Factor Eating Questionnaire, K—knowledge (EHQ), P—problems (EHQ), F&B—feelings and behaviors (EHQ), UE—Uncontrolled eating (TFEQ-R18), EE—Emotional eating (TFEQ-R18); * *p* < 0.05.

**Table 5:** Means, standard deviation, and Wilcoxon–Mann–Whitney statistics for study variables.

Measure	Female		Male		*U* (1, 965)	*p*-Value	*d*
	*M*	*SD*	*M*	*SD*			
EHQ	25.7	7.32	24.7	6.91	121732 *	0.038 ^a^	0.14
Knowledge (EHQ)	5.96	2.33	5.63	2.18	112933	0.990	
Problems (EHQ)	10.87	3.69	10.45	3.48	121099	0.050	0.17
Feelings and behaviors (EHQ)	3.14	3.09	8.62	3.00	124090 **	0.008 ^a^	
Body dissatisfaction (EDI)	1.41	0.49	1.38	0.49	115178	0.433	
Cognitive restraint (TFEQ-R18)	9.75	3.75	11.83	4.03	79207 ***	<0.001 ^a^	−0.54
Uncontrolled eating (TFEQ-R18)	10.22	−6.03	15.06	7.11	67251 ***	<0.001 ^a^	−0.75
Emotional eating (TFEQ-R18)	3.73	2.68	4.93	3.05	86818 ***	<0.001 ^a^	−0.43
Body mass index	22.26	3.76	22.85	3.79	101221 **	0.006 ^a^	−0.15
Age	23.86	5.77	22.62	3.34	6119016	0.147	

EHQ—Eating Habits Questionnaire; EDI—Eating Disorder Inventory; TFEQ-R18—Three-Factor Eating Questionnaire. ^a^—Numbers that indicate significant *p*-values: * *p* < 0.05; ** *p* < 0.01; *** *p* < 0.001.

## Appendix A

**Table A1:** The Polish version of the EHQ.

	Fałsz, Całkowita Nieprawda	Trochę Prawda	Głównie Prawda	Całkowita Prawda
1. Jestem lepiej niż inni poinformowany(a) w kwestii zdrowego odżywiania się	☐	☐	☐	☐
2. Odrzucam propozycje towarzyskie, które wiążą się z jedzeniem niezdrowego jedzenia	☐	☐	☐	☐
3. Sposób w jaki przygotowuję jedzenie jest ważny w mojej diecie	☐	☐	☐	☐
4. Ilość zasad obowiązujących w mojej diecie wzrosła	☐	☐	☐	☐
5. Moje nawyki żywieniowe są lepsze od nawyków żywieniowych innych ludzi	☐	☐	☐	☐
6. Rozpraszają mnie myśli dotyczące zdrowego odżywiania się	☐	☐	☐	☐
7. Jem tylko to, na co moja dieta pozwala	☐	☐	☐	☐
8. Moje zdrowe odżywianie się jest znaczącym źródłem stresu w moich relacjach z innymi ludźmi	☐	☐	☐	☐
9. Podejmowałem(am) wysiłki, by z biegiem czasu jeść zdrowiej	☐	☐	☐	☐
10. Moja dieta ma wpływ na rodzaj zatrudnienia jakie bym podjął/podjęła	☐	☐	☐	☐
11. Moja dieta jest lepsza niż dieta innych ludzi	☐	☐	☐	☐
12. Czuję, że mam kontrolę kiedy zdrowo jem	☐	☐	☐	☐
13. W ciągu ostatniego roku przyjaciele lub członkowie rodziny powiedzieli mi, że zbytnio przejmuję się zdrowym odżywianiem	☐	☐	☐	☐
14. Mam trudność ze znalezieniem restauracji serwujących jedzenie, które jem	☐	☐	☐	☐
15. Jedzenie w taki sposób, w jaki ja to robię, daje mi poczucie satysfakcji	☐	☐	☐	☐
16. Niewiele pokarmów jest dla mnie zdrowych	☐	☐	☐	☐
17. Rzadziej wychodzę z domu odkąd zacząłem/zaczęłam się zdrowo odżywiać	☐	☐	☐	☐
18. Spędzam więcej niż trzy godzinny dziennie na myśleniu o zdrowym jedzeniu	☐	☐	☐	☐
19. Czuję się świetnie, kiedy zdrowo jem	☐	☐	☐	☐
20. Sztywno trzymam się diety opartej na zdrowym jedzeniu	☐	☐	☐	☐
21. Przygotowuję jedzenie w najzdrowszy możliwy sposób	☐	☐	☐	☐
